# Identifying possible reasons why female street sex workers have poor drug treatment outcomes: a qualitative study

**DOI:** 10.1136/bmjopen-2016-013018

**Published:** 2017-03-22

**Authors:** Nikki Jeal, John Macleod, Chris Salisbury, Katrina Turner

**Affiliations:** 1 Centre for Academic Primary Care School of Social & Community Medicine Bristol, University of Bristol, Bristol, UK; 2 The National Institute for Health Research Collaboration for Leadership in Applied Health Research and Care West (NIHR CLAHRC West) University Hospitals Bristol NHS Foundation Trust, Bristol, UK

**Keywords:** sex work, QUALITATIVE RESEARCH

## Abstract

**Aims:**

To explore street sex workers (SSWs) views and experiences of drug treatment, in order to understand why this population tend to experience poor drug treatment outcomes.

**Design:**

In-depth interviews.

**Setting:**

Bristol, UK.

**Participants:**

24 current and exited SSWs with current or previous experience of problematic use of heroin and/or crack cocaine.

**Findings:**

Participants described how feeling unable to discuss their sex work in drug treatment groups undermined their engagement in the treatment process. They outlined how disclosure of sex work resulted in stigma from male and female service users as well as adverse interactions with male service users. Participants highlighted that non-disclosure meant they could not discuss unresolved trauma issues which were common and which emerged or increased when they reduced their drug use. As trauma experiences had usually involved men as perpetrators participants said it was not appropriate to discuss them in mixed treatment groups. SSWs in recovery described how persistent trauma-related symptoms still affected their lives many years after stopping sex work and drug use. Participants suggested SSW-only services and female staff as essential to effective care and highlighted that recent service changes were resulting in loss of trusted staff and SSW-only treatment services. This was reported to be reducing the likelihood of SSWs engaging in drug services, with the resultant loss of continuity of care and reduced time with staff acting as barriers to an effective therapeutic relationship.

**Conclusions:**

SSWs face many barriers to effective drug treatment. SSW-only treatment groups, continuity of care with treatment staff and contact with female staff, particularly individuals who have had similar lived experience, could improve the extent to which SSWs engage and benefit from drug treatment services. Service engagement and outcomes may also be improved by drug services that include identification and treatment of trauma-related symptoms.

**Strengths and limitations of this study**

- This appears to be the first study to provide a unique overview of the multiple influences of service provision on street sex workers (SSWs) treatment outcomes through a detailed exploration of the views and experiences of SSWs in relation to drug services.
- Although drug-dependent SSWs can be a challenging population to research, this study includes the views of 24 SSWs and reached data saturation.
- The purposive sample of participants ensured a broad spectrum of experience and opinion was captured by including SSWs whose drug use ranged from daily use to no use in the past 4 years.
- This is the first study to detail how trauma-related experiences affect drug use and use of drug services among SSWs.

## Introduction

The poor health of sex workers and the risks they face continue to be a source of international concern.^1–3^ Sex work is frequently linked with problematic drug use^4–6^ and drug-dependent sex workers typically work on the street,^7–9^ experiencing the greatest risks to health.^10^

Drug dependency underpins much of the morbidity this group experience.^11^ Behavioural effects of drug use or withdrawal symptoms may reduce ability to negotiate condom use or safe working location and increase risk-taking while working.^12^ ^13^ Injection drug use exposes street sex workers (SSWs) to risks of bloodborne virus infection, abscesses at injecting sites, deep vein thrombosis and septicaemia.^14^ This is in addition to the health risks of street sex working which include genital infection,^15^ bloodborne infection,^16^ poor mental health,^17^ ^18^ exposure to violence^19^ and an increased risk of death.^20^

Drug dependency reinforces involvement in sex work^21^ and SSWs report feeling trapped in a ‘work-score-use’ cycle.^22^ The likelihood of stopping sex work is inversely related to injecting drug use^23^ and use of drug services and involvement in sex work often run a relapsing and remitting course.^24–26^

The most frequent drugs of abuse for SSWs are heroin and crack cocaine which are associated with the poorest outcomes from treatment.^27^ Compared with other problematic drug users, SSWs' drug use is more prolific,^28^ they are less successful in achieving abstinence^29^ and have a higher drug-related mortality rate. Women involved in sex work tend to have initiated problem drug use at younger ages and inject drugs for more years than non-sex-working female service users.^30^

These longstanding problems^31^ ^32^ suggest that there may be sex worker-specific issues that current services are not addressing. Stigma has been highlighted as an issue affecting sex workers who access drug services.^33^ ^34^ Poor mental health has been implicated in SSWs' poor outcomes from drug treatment services.^33^ Experience of abuse and violence from young age^35^ ^36^ something frequently reported by SSWs,^37^ has also been linked to poor treatment outcomes in women.^38^

Such findings have led to recommendations that SSWs receive drug treatment services that include psychological support,^39^ mental healthcare^40^ and trauma-informed services.^41^ However, clear guidance on which interventions will be most effective for SSWs or how different elements of service delivery could improve outcomes for SSWs is lacking. The aim of this study was to explore SSWs' views and experiences of drug treatment services in order to identify factors that affect their engagement with, and response to, drug treatment. These findings are needed to inform development of effective interventions and services development that improve treatment outcomes in this group.

## Methods

To be eligible for inclusion, individuals needed to have more than 3 months current or previous experience of street sex work and heroin and/or crack cocaine use in the previous 5 years, and be aged 18 years and over at the time of interview. Flyers advertising the research were left at five establishments known to provide services used by SSWs working in Bristol. Eligibility was assessed through completion of a screening questionnaire which was completed by women who contacted the researcher in order to take part. None of those who were eligible declined to take part. A purposeful sampling method was used to ensure that in-depth interviews were held with women whose drug use varied from daily drug use to no drug use in the past 12 months. Interviews were employed as they would enable participants' views and their experiences of drug services to be explored in detail from their own perspective,^42^ and would allow participants to raise issues that were salient to them. Each potential interviewee was provided with a participant information sheet at the time of recruitment. Immediately prior to interview, the researcher went through the information sheet with the participant, answered any questions she had and secured written consent from the individual to be interviewed.

Interviews were undertaken by NJ at the recruiting site, at the University, or in the participant's own home. NJ was introduced to participants as a doctor and as a researcher. Owing to her clinical work and previous research projects, she was known to some of the women she interviewed. The interviews lasted between 20 and 90 min. A topic guide was used to ensure consistency across interviews and included questions about experience of treatment and care, and how they affected levels of drug use, specific treatment needs of SSWs and suggestions for service improvement, as well as awareness of and experiences resulting from recent changes on drug treatment services. With participant consent, the interviews were audio-taped and transcribed verbatim. Each respondent received a £20 shopping voucher to thank them for their time.

NJ and KT read and re-read eight interview transcripts which had been purposefully sampled to ensure they included transcripts from across the spectrum of interviews held, for example, from women still using drugs to women who had not used drugs for over 12 months. The two researchers coded the transcripts and then met to discuss their coding and themes they had identified. A coding frame was drafted during this discussion. It included codes which reflected themes identified and areas explored in the topic guide. Related codes were grouped under headings which reflected overarching themes, for example, service changes and service suggestions were grouped under drug service use. NJ applied the coding frame to three of the transcripts and, confident it was appropriate, then uploaded all the transcripts into the software package NVivo and electronically coded each one. An approach based on Framework analysis^43^ was then used to summarise the data. This entailed summarising the data in a table where each row represented a participant and each column heading was based on the codes we had developed, rather than predefined headings, which is usually the case when using Framework analysis. Doing this enabled comparisons to be made within and across the data and for overall themes and deviant cases to be identified.

## Results

### Participants

Twenty-four participants were interviewed between May and July 2014 (table 1).

**Table 1:** Participant summary—grouped by frequency of drug use

	Age (years)	Time since street drugs	Time since substitute	Longest clean (off everything)	How often using street drugs now	Main route when last used	Time since worked	Total worked
Daily use								
1	51	1 day	Never (allergy)	1 month	X1/day	Smoke	1 day	31 years
2	52	1 day	NA (amphetamine and crack)	6 months	Every day	Smoke	2 days	40 years
3	32	Today	Current	1 year	X5–20/day	IDU	1 day	15 years
4	49	Today	Current	7 years	X10/day	IDU	5 days	3 years+
5	27	Today	Current	19 weeks	X3–4/day	Smoke	Today	7 years
6	26	Today	Current	4 years	X10/day	IDU	2 days	1 year
7	52	1 day	>1 year	1 year	>Once/day	Smoke	5 days	35 years
8	54	1 day	Current	4 months	X7/day	IDU	6 months	10 years
9	38	Today	Current	Always OST	X10/day	IDU	1 day	1½ years
Regular use								
10	50	1 day	Current	8 years	X1–2/week	Smoke	2 months	14 years
11	49	Today	Current	3½ years	X2–3/week	Smoke	2 months	20 years
12	36	1 day	Current	2 weeks	Alternate days	IDU	1 month	4 years
13	46	2 weeks	2 years	3½ years	X1/month	Smoke	3 years	32 years
14	41	2 days	Current	6 months	X1–2/week	IDU	2 days	5 years
15	34	2 days	Current	2 weeks	X2/week	IDU	2 days	20 years
Problem alcohol use								
16	38	1 month	Current	Never	Seasonal	Smoke	5 months	10 years
17	40	1 day	Current	Few days	When misses methadone	IDU	2 days	10 years
In recovery								
18	40	4 years	3½ years	4 years	−	Smoked	5 years	12 years
19	41	13 months	13 months	13 months	−	IDU	13 months	15 years
20	41	2 years	13 months	14 months	−	IDU	2 years	20 years
21	36	2½ years	2 years	2 years	−	IDU	2½ years	15 years
22	44	2 years	3 years	2 years	−	IDU	7 years	10 years
23	37	3¾ years	3½ years	3½ years	−	IDU	3½ years	1 year
24	37	4 years	4 years	4 years	−	Smoke	4 years	8 years

IDU, injecting drug use; NA, not available; OST, opiate substitution treatment.

Participants were aged between 26 and 54 years. All participants disclosed experience of street sex work and dependency on heroin and/or crack cocaine in the previous 5 years. The majority (14) of participants had injected drugs at most recent use. Participants had lived and worked in a variety of towns and cities, but their most recent or current episode of sex working had been in Bristol. Despite sampling for maximum variation, there were no clear differences between the accounts of daily users, regular users and women who were in recovery, or between women who varied, for example, in relation to their age or time working as an SSW.

Nine participants, six of whom were taking prescribed methadone, reported using illicit drugs daily (daily use). Their reported use ranged from once a day to up to 20 times a day. Seven participants, six of whom were on prescribed methadone, said they used less than daily (regular use). Reported frequency of drug use ranged from every second day to once a month. Seven participants described themselves as in recovery and had not used prescribed substitutes or illicit drugs for between 13 months and 4 years (in recovery). Two women, both on prescribed methadone, described that alcohol had replaced heroin and crack as their main dependency (problem alcohol use).

All but two of the participants who were in active addiction at the time of interview described having been in recovery in the past. Their reported time in recovery ranged from a few days to 8 years.

### Drug service use

Participants were recruited from a single location (a charity that provides support and advocacy for SSWs) but each participant described having used a variety of drug treatment services and therapies, in and out of Bristol, and based in community and residential settings.

#### Talking therapies

##### Group work

All the participants described group work as a central part of drug treatment. Groups were portrayed as having a potentially positive treatment role but participants said that for SSWs, their usefulness was limited as SSWs felt unable to talk about sex work. Though sex work was a large part of their life, their identity and their drug use, they said they did not want to discuss it because of the negative behaviour of male and female service users towards SSWs.

RES: I did used to get treated different because I worked…I was stigmatised for it. Yeah.

INT: Yeah. And did that make going to the groups hard?

RES: Yeah, a lot of shame was in that. Yeah.

Participant 24, in recovery.

The majority of participants commented that disclosing sex work encouraged negative attention from male service users. Participants described becoming the focus of unwanted sexual advances, particularly from those on criminal justice-driven treatment packages. This led to participants needing to constantly deflect unwanted advances or risk becoming involved in damaging relationships, with both activities undermining their treatment progress. Relationships were reported to frequently result in premature termination of treatment and could ultimately lead them back to drug use.

RES: I was only in there like a few days and I was with one of the kiddies in there, so already I was distracted…I ended up going using…I left him as soon as I got outside.

Participant 24, in recovery

Participants also described how mixed treatment groups increased their likelihood of sex working by facilitating access to relationships with men who could act as their protection while working.

It's a two way street because I know that I saw men and I thought (laughs), I can manipulate them, it's easy, I've got everything he wants…I can go and keep him in drugs [by selling sex] and he can protect me.

Participant 22, in recovery

One woman described how a timely move from a mixed residential rehab to an all-female one had been crucial to her continuing into recovery.

RES: there was one lad that I quite liked…but, [the drugs worker from the sex work support and advocacy charity] quickly shuffled me into [a female residential rehab], all female, and I needed it.

Participant 23, in recovery

Not being able to talk about sex work in groups was also described as preventing participants from talking about past trauma and abuse. This was viewed as problematic as adverse psychological or emotional symptoms had a significant impact on their lives and drug use. Men had generally been the perpetrators of the trauma which meant they could not talk about it in front of them.

RES: Men just abuse me left, right and centre, I was beaten silly, I was raped, the guy got ten years for what he done to me…I had a pimp when I was twenty one, he beat me, stabbed me, bit me, you know, beat me with baseball bats, bottles, you know, it was horrendous, and some of it's so traumatic, you know, to share that in front of a man.

Participant 20, in recovery

This meant that experiences of abuse and trauma often went undiscussed, unidentified and untreated. One participant described surviving a serious and traumatic assault for which she had received no support.

RES: About there [near drug services] [I was] beat up and dropped out of a fifteenth floor flat's balcony. Landed in a…lucky I'm skinny and underweight because I'd have been dead. I went down two fucking flights of, two floors from the top to two floors down, landed in a scaffolding net, the safety net. Thank God fucking…the bloke on the balcony, poor soul, he had to have counselling because he was so frightened by what he seen and he says he was just terrified…I was lucky. He said don't move the safety net. He said lucky you are not any fatter than what you are because you would have gone straight through. But I didn't have any counselling [to help manage the psychological effects of that experience].

Participant 2, regular user

##### One-to-one sessions

The majority of participants described one-to-one sessions and how they were an important part of dealing with addiction, as they represented an opportunity to go deeper into personal issues than was possible in groups. Participants described staff gender as an issue for SSWs who were likely to find it difficult to be alone with a man. However, one participant described sessions with a male counsellor at the start of her drug treatment which made her feel like she had been ‘raped again’ but then a positive experience later into her recovery, suggesting something may have changed.

They gave me a male counsellor because I was quite anti male, yeah. Em, I felt, I know, it was really weird, I was like that, are you kidding me? Anyway, as it happens, he was the nicest guy and he was, he was so (sighs) I can't really, er, it did, it worked, it actually really worked.

Participant 22, in recovery

##### Substitute prescribing

All but one participant described having received substitute prescribing through their general practitioner (GP) and/or through specialist drug services. Those attending the GP surgery either saw their GP at each visit or had ‘shared care’ where they saw a drug worker who provided support and gave them the prescription for opiate substitutes prescribed by the GPs.

Women who received methadone from a GP they had been with for a long time spoke positively about that service. Other participants described using a string of GPs, and said they had been able to manipulate the practitioner by playing on their naiveté and/or lack of knowledge about their prescribing history in order to receive large amounts of medications for misuse.

RES: I could manipulate…I was on ridiculous scripts for doctors at different times…I remember once, I got, you know, given a script, I come out of the doctors and, em, I would have carrier bags full of this stuff [opiate substitutes].

Participant 23, in recovery

Participants said that specialist prescribing services were less easy to manipulate but that mixed morning dispensing was problematic for SSWs.

RES:…like sometimes when you’ve been out all night [sex working] and you're a mess you don't want to go in and sit and wait in front of men and…It's embarrassing.

Participant 1, daily user

Participants who saw drug workers said that they did not see the workers frequently enough, the appointments with them were too short and staff turnover was high, which meant they felt unable to develop the trust they needed to develop a therapeutic relationship.

RES: Depending on the worker as well, depending on, again I was quite lucky that the person, I've had a few shared care workers but that's not good chopping and changing the workers. Because it is all about trust, you build up a relationship with somebody and then there's somebody else there.

Participant 10, regular user

Participants said they found staff that were ex-service users had a better understanding of the issues they faced which gave their guidance more validity. These members of staff were also described as having potential to give service users hope of recovery.

RES: people that are going through the same stuff, or that have been through the same stuff, that have recovered and said look at me, you can do it, but not being so hypocritical if you know what I mean, just saying come on, yeah.

Participant 6, daily user

### SSW-specific needs and suggestions to improve engagement

When asked about SSW-specific needs in drug services, almost all participants focused on the need for sex workers to feel safe, not judged and able to address experiences of sex working in order to engage.

They outlined how they needed SSW-only groups so they could openly discuss their sex-working experiences and improve the effectiveness of group work.

RES: I mean probably just because I am a woman as well I think being female and having worked, I think it is quite good, important to talk with other working girls, you know girls that have had the same similar experiences. You have got to have identification, it's no good if you haven't got any identification with anybody.

Participant 10, regular user

Participants also outlined the need to address the ongoing psychological effects of traumatic experiences which were difficult to manage when drugs were reduced.

INT: Do you think that women who have worked on the street have got different service needs to other people?

RES: Yeah, definitely. I think they've got more extensive like…a lot of them will have had like sexual trauma, some kind of sexual abuse and I think that can make it hard to stop using because it comes back and…it's like a post traumatic stress disorder…I never used to believe in panic attacks but it's like a physical, you get a real physical sense of…it's horrendous, really frightening.

Participant 3, daily user

These symptoms were described as a long-term problem. Some participants described use of medication but all the participants in recovery, who avoided mind-altering substances for fear of relapse, said that past experiences still affected them, even when they had not sex worked for a number of years.

RES: The shame around that can still affect me today and, um, it was massive in my sexual relationships because I just associated that with working…the one [partner] I had before…it was just like there was no connection. I was still quite dead when it come to that and it was like, I did it because…I did want to but it was still a more…there was no connection there and it was more… reminded me of working, I know there's some girls and a few of my friends as well, some of them are like 8 years clean, 7 years clean and it still affects them and their sexual relationships, you know. It's, um, quite traumatising. Um, yeah.

Participant 24, in recovery

Many participants mentioned the importance of having female staff in order to be able to openly discuss the trauma.

RES: It's not just the drugs that have done the damage by then, it's the, you know, sex with strangers…the violence on the street…Constant, possibly a pimp, physical abuse on a daily basis. And self loathing, you know, I mean, it's bad enough when you just use drugs, when you've had to sell your body to do it, you know.

INT: And so do you think there's particular support needs then, over and above what would be your average drug services?

RES: Definitely. And female…

Participant 22, in recovery

### Effects of recent local drug service changes

Participants were asked about their views and experiences of recent changes to drug services in Bristol. They described how changes seemed to be driven by spending cuts and how they had noticed loss of staff they knew and trusted. This was described as a source of stress and was resulting in SSWs not going to drug services and undertaking more risky drug use behaviour.

INT: So you've noticed changes.

RES: All closed yeah…Like people's losing their jobs man, like counsellors and drug…It's awful, we are lost without them.

INT: And have you noticed that it is better or worse than before?

RES: It is worse now. People are just not even bothering to go down to get exchanges in needles, just use them off the streets…because people they are losing the people that they've gone and seen all the time to go, trusted, go and see and picked up needles and not feel they are going to go to the police, or got a copper waiting outside. So all these people are just losing their jobs and it's so sad, because they, it takes so long to trust someone in the system and then you got to lose them haven't you? It's like losing a family. It's sad because you think they were doing a good job they were, I was getting on with they, they were helping me and all of a sudden bang, you're back to square one again. The next thing you know, so and so's leaving, they ain't got enough funding, what's it all for?

Participant 2, daily user

Loss of staff was also said to be increasing the workloads of those who were left, which lengthened waiting lists.

RES:, it'll be nearly a month now to get onto my, the shared care worker that I had before. He's so overloaded with work and stuff.

Participant 10, regular user

Participants said that cuts in funding were also resulting in loss of services specifically for sex workers, either through being opened up to addicts who were not sex workers or through services being shut down. Participants noted that services merging to save money affected the quality of the services. The ‘tick box’ approach was characterised as less caring, something that was difficult to measure but central to effectiveness.

RES: Some of these places [drug services] are beginning to really lose their heart and soul and they're just becoming almost like faceless, uncaring, things that, “Oh, the government does provide but” you know, and that they are there, but actually they've thrown a bit of money at things and tried to get other things to amalgamate and then it becomes something that it's not and then it kind of all fragments and it's kind of all things are being lost that really matter…I know the combination of things is actually generally down to trying to save money and trying to shrink things in order to help more people…But it's losing the most important thing which is heart and soul and care.

Participant 11, regular user

## Discussion

### Principal findings

A history of sex working raised particular issues for SSWs in drug treatment services. They felt unable to disclose sex working in order to avoid being stigmatised by male and female group members as well as avoiding relationships that undermined their engagement in treatment. Mixed gender drug services and groups also prevented them from discussing trauma-related issues in front of male group members and male staff due to experience of men as perpetrators. Additionally some perpetrators were also service users. Unidentified and untreated trauma-related morbidity continued to affect participants who were no longer using drugs or sex working. SSW-only groups with female staff were suggested as a solution.

Drug service cuts were noted to be resulting in loss of SSW-only services and loss of trusted staff which discouraged service engagement. Loss of staff also resulted in longer wait times to be seen and shorter appointments, further reducing engagement and undermining care. Continuity of care and more time to engage with workers, particularly those with similar lived experience, was reported to improve service benefit and outcomes.

### Strengths and weaknesses

SSWs can be difficult to engage with research but we managed to interview over 20 women and to reach data saturation. A purposeful sampling method ensured that participants with a range of experiences of drug use were interviewed, which allowed comparison of contrasting views across different groups. Despite leaving flyers at a number of premises participants were recruited through a single location. However, it was apparent during interviews that participants had accessed a number of different services of different types so were well placed to discuss a broad range of service options. As participants knew the lead researcher was a medical doctor, they may have been reluctant to describe behaviours that might be perceived as negative. However, experience of working with this population for over a decade has resulted in building trust which allowed discussion of sensitive subjects in depth. To reduce biases introduced by the researcher, the study's focus, the topic guide and what data should be collected were all discussed with the other authors. In addition, the coding frame was developed through discussions with a second researcher, following independent coding of transcripts.

### Implications

Previous research has highlighted the difficulties encountered by women in mixed gender drug treatment services.^44^ Our research highlights the additional unique challenges faced by sex workers due to their potential for commercially fuelled relationships and stigmatisation from male and female service users due to their sex-working history. Women-only groups have been advocated but our findings go further by supporting the need for SSW-only drug treatment.^33^

Our findings are consistent with previous work that reports trauma-related morbidity affecting female drug users^45^ including SSWs.^18^ ^35^ ^36^ Women-only trauma-informed programmes designed to reduce levels of drug use have had inconclusive results,^46^ ^47^ though some have suggested that certain subgroups^48^ ^49^ such as sex workers,^50^ may benefit from this approach. Our findings are in keeping with theory suggesting that drug use is a strategy to avoid traumatic symptoms.^51^ Failure to adequately address trauma when attempting to reduce drug use in this group may underpin the lack of effective drug treatment for SSWs.^52^ There are no SSW-specific integrated treatment studies to date and our findings highlight the long-term effects of untreated trauma on SSWs and the need for future research in this area.

While our study provides some of the arguments for SSW-only drug treatment facilities, these are likely to be costly to deliver. However, mixed gender programmes are not cost-effective for women^53^ and an effective drug service could reduce indirect costs associated with SSWS' drug use, for example, time in prison and future healthcare costs. This evidence is much needed as currently specialist services are being rationalised and SSW-only services are being lost.^54^ Our findings demonstrate the negative impact that such loss of specialist staff and service is having on SSW drug use and service use.

## Conclusions

Accounts from SSWs demonstrated how their experiences of sex work prevented them from fully engaging and benefitting from current drug services. The reactions of other service users, as well as SSWs feeling unable to openly discuss the issues arising from their work, contribute to poor treatment outcomes. Recent service rationalisation and loss of specialist services are adding to these problems. Participants suggested that SSW-only services with specialist staff, and ideally with continuity of care, are needed for drug services to meet the needs of SSWs. Identification and treatment of comorbid trauma-related symptoms may facilitate better engagement in treatment and facilitate long-term recovery.

Sustained reduction in illicit drug use is central to effective treatment of the excess physical and psychological morbidity and increased mortality experienced by SSWs. The multiple and unique effects of sex working on SSWs' ability to engage with, and fully benefit from, drug services need to be acknowledged and addressed. Our findings suggest that the effectiveness of drug treatment services for SSWs could be improved significantly if they were.
